# (*E*)-3-(2-Chloro­phen­yl)-1-(4-chloro­phen­yl)prop-2-en-1-one

**DOI:** 10.1107/S1600536808015420

**Published:** 2008-05-30

**Authors:** Hoong-Kun Fun, Samuel Robinson Jebas, Ibrahim Abdul Razak, P. S. Patil, S. M. Dharmaprakash, E. Deepak D’Silva

**Affiliations:** a X-ray Crystallography Unit, School of Physics, Universiti Sains Malaysia, 11800 USM, Penang, Malaysia; b Department of Studies in Physics, Mangalore University, Mangalagangotri, Mangalore 574 199, India

## Abstract

The title compound, C_15_H_10_Cl_2_O, adopts an *E* configuration with respect to the C=C bond of the propenone unit. The dihedral angle between the two benzene rings is 32.4 (1)°. Intra­molecular C—H⋯O and C—H⋯Cl hydrogen bonds generate an *S*(5)*S*(5)*S*(5) motif. In addition, the crystal structure is stabilized by weak inter­molecular C—H⋯O hydrogen bonds.

## Related literature

For related literature on chalcones, see: Fun *et al.* (2007 ▶); Patil *et al.* (2007 ▶). For bond-length data, see: Allen *et al.* (1987 ▶). For graph-set motifs, see: Bernstein *et al.* (1995 ▶). 
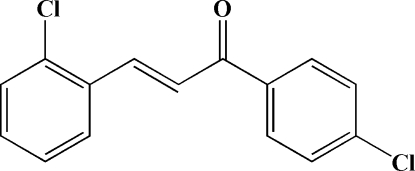


## Experimental

### 

#### Crystal data

C_15_H_10_Cl_2_O
*M* *_r_* = 277.13
Orthorhombic, 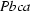

*a* = 7.2777 (1) Å
*b* = 11.2686 (2) Å
*c* = 30.2365 (6) Å
*V* = 2479.68 (7) Å^3^
*Z* = 8
Mo *K*α radiation
μ = 0.51 mm^−1^
*T* = 100.0 (1) K
0.51 × 0.34 × 0.19 mm

#### Data collection

Bruker SMART APEX2 CCD area-detector diffractometer
Absorption correction: multi-scan (*SADABS*; Bruker, 2005 ▶) *T*_min_ = 0.781, *T*_max_ = 0.911
42713 measured reflections
5996 independent reflections
4413 reflections with *I* > 2σ(*I*)
*R*_int_ = 0.042

#### Refinement

*R*[*F* ^2^ > 2σ(*F* ^2^)] = 0.045
*wR*(*F* ^2^) = 0.127
*S* = 1.05
5996 reflections
163 parameters
H-atom parameters constrained
Δρ_max_ = 0.55 e Å^−3^
Δρ_min_ = −0.21 e Å^−3^

### 

Data collection: *APEX2* (Bruker, 2005 ▶); cell refinement: *APEX2*; data reduction: *SAINT* (Bruker, 2005 ▶); program(s) used to solve structure: *SHELXTL* (Sheldrick, 2008 ▶); program(s) used to refine structure: *SHELXTL*; molecular graphics: *SHELXTL*; software used to prepare material for publication: *SHELXTL* and *PLATON* (Spek, 2003 ▶).

## Supplementary Material

Crystal structure: contains datablocks global, I. DOI: 10.1107/S1600536808015420/lh2627sup1.cif

Structure factors: contains datablocks I. DOI: 10.1107/S1600536808015420/lh2627Isup2.hkl

Additional supplementary materials:  crystallographic information; 3D view; checkCIF report

## Figures and Tables

**Table 1:** Hydrogen-bond geometry (Å, °)

*D*—H⋯*A*	*D*—H	H⋯*A*	*D*⋯*A*	*D*—H⋯*A*
C7—H7*A*⋯Cl1	0.93	2.65	3.0484 (12)	107
C7—H7*A*⋯O1	0.93	2.46	2.7973 (15)	101
C15—H15*A*⋯O1	0.93	2.46	2.7691 (15)	100
C12—H12*A*⋯O1^i^	0.93	2.59	3.2064 (16)	125

Symmetry code: (i) 
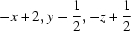
.

**Fig. 1.**
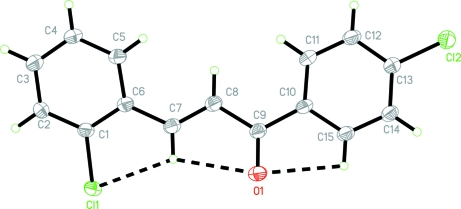
The molecular structure of the title compound, showing 50% probability displacement ellipsoids and the atom numbering scheme. Intramolecular H-bonds are shown as a dashed lines.

**Fig. 2.**
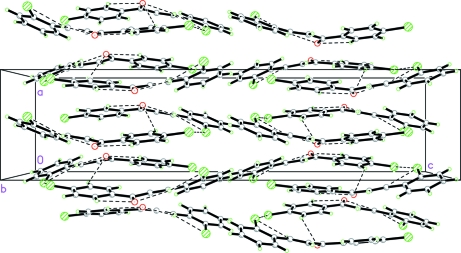
The crystal packing of the title compound, viewed along the b axis.

## supplementary crystallographic information

### Comment

In continuation of our study on chalcone derivatives (Fun *et al.*, 2007; Patil *et al.*, 2007), the crystal structure of the title compound (I) is reported herein.

In (I), the molecule exhibits an E configuration with respect to the C8═C9 double bond with the C7–C8–C9–C10 torsion angle being 163.0 (2) °. The bond lengths in the title compound (Fig. 1) have normal values (Allen *et al.*, 1987). The dihedral angle between the two benzene rings is 32.7 (1)°.

Intramolecular C—H···O  and C-H···Cl hydrogen bonds generate an S(5)S(5)S(5) motif (Bernstein *et al.*, 1995). In addition, the crystal structure is stabilized by weak intermolecular C—H···O hydrogen bonds (Table 1).

### Experimental

The compound (I) was synthesized by the condensation of 2 -chlorobenzaldehyde (0.01 mol) with 4-chloroacetophenones (0.01 mol) in methanol (60 ml) in the presence of a catalytic amount of sodium hydroxide solution (5 ml, 30%). After stirring (6 h), the contents of the flask were poured into ice-cold water (500 ml) and left to stand for 12 h. The resulting crude solid was filtered and dried. Crystals suitable for single-crystal X-ray diffraction were grown by slow evaporation of an acetone solution at room temperature.

### Refinement

H atoms were positioned geometrically [C—H = 0.93 Å] and refined using a riding model, with *U*_iso_(H) = 1.2*U*_eq_(C).

### Crystal data

**Table:**

C_15_H_10_Cl_2_O	*F*_000_ = 1136
*M**_r_* = 277.13	*D*_x_ = 1.485 Mg m^−^^3^
Orthorhombic, *P**b**c**a*	Mo *K*α radiation λ = 0.71073 Å
Hall symbol: -P 2ac 2ab	Cell parameters from 8869 reflections
*a* = 7.2777 (1) Å	θ = 2.7–36.0º
*b* = 11.2686 (2) Å	µ = 0.51 mm^−^^1^
*c* = 30.2365 (6) Å	*T* = 100.0 (1) K
*V* = 2479.68 (7) Å^3^	Block, colourless
*Z* = 8	0.51 × 0.34 × 0.19 mm

### Data collection

**Table:**

Bruker SMART APEX2 CCD area-detector diffractometer	5996 independent reflections
Radiation source: fine-focus sealed tube	4413 reflections with *I* > 2σ(*I*)
Monochromator: graphite	*R*_int_ = 0.042
*T* = 100.0(1) K	θ_max_ = 36.4º
φ and ω scans	θ_min_ = 1.4º
Absorption correction: multi-scan(SADABS; Bruker, 2005)	*h* = −7→12
*T*_min_ = 0.781, *T*_max_ = 0.911	*k* = −17→18
42713 measured reflections	*l* = −50→48

### Refinement

**Table:**

Refinement on *F*^2^	Secondary atom site location: difference Fourier map
Least-squares matrix: full	Hydrogen site location: inferred from neighbouring sites
*R*[*F*^2^ > 2σ(*F*^2^)] = 0.045	H-atom parameters constrained
*wR*(*F*^2^) = 0.127	*w* = 1/[σ^2^(*F*_o_^2^) + (0.0601*P*)^2^ + 0.6003*P*] where *P* = (*F*_o_^2^ + 2*F*_c_^2^)/3
*S* = 1.05	(Δ/σ)_max_ < 0.001
5996 reflections	Δρ_max_ = 0.55 e Å^−^^3^
163 parameters	Δρ_min_ = −0.21 e Å^−^^3^
Primary atom site location: structure-invariant direct methods	Extinction correction: none

### Special details

**Table:**

Experimental. The data was collected with the Oxford Cyrosystem Cobra low-temperature attachment.
Geometry. All e.s.d.'s (except the e.s.d. in the dihedral angle between two l.s. planes) are estimated using the full covariance matrix. The cell e.s.d.'s are taken into account individually in the estimation of e.s.d.'s in distances, angles and torsion angles; correlations between e.s.d.'s in cell parameters are only used when they are defined by crystal symmetry. An approximate (isotropic) treatment of cell e.s.d.'s is used for estimating e.s.d.'s involving l.s. planes.
Refinement. Refinement of *F*^2^ against ALL reflections. The weighted *R*-factor *wR* and goodness of fit *S* are based on *F*^2^, conventional *R*-factors *R* are based on *F*, with *F* set to zero for negative *F*^2^. The threshold expression of *F*^2^ > σ(*F*^2^) is used only for calculating *R*-factors(gt) *etc*. and is not relevant to the choice of reflections for refinement. *R*-factors based on *F*^2^ are statistically about twice as large as those based on *F*, and *R*- factors based on ALL data will be even larger.

### Fractional atomic coordinates and isotropic or equivalent isotropic displacement parameters (Å^2^)

**Table:**

	*x*	*y*	*z*	*U*_iso_*/*U*_eq_	
Cl1	0.92678 (5)	0.49110 (3)	0.058017 (10)	0.02768 (8)	
Cl2	1.06791 (4)	0.29033 (3)	0.406266 (11)	0.02816 (8)	
O1	1.19326 (15)	0.53008 (8)	0.20697 (3)	0.0293 (2)	
C1	0.99869 (17)	0.34415 (10)	0.06050 (4)	0.0217 (2)	
C2	0.9725 (2)	0.27399 (11)	0.02335 (4)	0.0261 (2)	
H2A	0.9233	0.3066	−0.0023	0.031*	
C3	1.0200 (2)	0.15500 (11)	0.02468 (5)	0.0295 (3)	
H3A	1.0032	0.1074	−0.0001	0.035*	
C4	1.0934 (2)	0.10670 (11)	0.06340 (5)	0.0277 (3)	
H4A	1.1250	0.0268	0.0644	0.033*	
C5	1.11899 (19)	0.17750 (10)	0.10012 (4)	0.0247 (2)	
H5A	1.1682	0.1443	0.1257	0.030*	
C6	1.07237 (16)	0.29880 (10)	0.09989 (4)	0.0214 (2)	
C7	1.10257 (17)	0.37394 (10)	0.13844 (4)	0.0229 (2)	
H7A	1.1007	0.4555	0.1337	0.028*	
C8	1.13268 (18)	0.33788 (10)	0.17986 (4)	0.0245 (2)	
H8A	1.1364	0.2572	0.1863	0.029*	
C9	1.16008 (17)	0.42575 (10)	0.21552 (4)	0.0226 (2)	
C10	1.14122 (17)	0.38733 (10)	0.26259 (4)	0.0212 (2)	
C11	1.10474 (19)	0.27011 (11)	0.27512 (4)	0.0258 (2)	
H11A	1.0948	0.2116	0.2536	0.031*	
C12	1.08325 (18)	0.24012 (11)	0.31927 (5)	0.0260 (2)	
H12A	1.0583	0.1622	0.3274	0.031*	
C13	1.09944 (16)	0.32798 (10)	0.35122 (4)	0.0227 (2)	
C14	1.13629 (18)	0.44474 (11)	0.33986 (4)	0.0247 (2)	
H14A	1.1471	0.5028	0.3616	0.030*	
C15	1.15668 (18)	0.47343 (10)	0.29565 (4)	0.0239 (2)	
H15A	1.1812	0.5516	0.2878	0.029*	

### Atomic displacement parameters (Å^2^)

**Table:**

	*U*^11^	*U*^22^	*U*^33^	*U*^12^	*U*^13^	*U*^23^
Cl1	0.03936 (18)	0.01927 (12)	0.02440 (15)	0.00338 (11)	0.00267 (12)	0.00341 (9)
Cl2	0.03176 (16)	0.02708 (15)	0.02565 (15)	−0.00451 (12)	0.00190 (11)	−0.00078 (10)
O1	0.0399 (5)	0.0181 (4)	0.0299 (5)	−0.0008 (4)	−0.0062 (4)	0.0006 (3)
C1	0.0255 (5)	0.0174 (4)	0.0223 (5)	−0.0004 (4)	0.0031 (4)	0.0014 (4)
C2	0.0355 (6)	0.0228 (5)	0.0201 (5)	−0.0030 (5)	0.0029 (5)	0.0015 (4)
C3	0.0414 (7)	0.0213 (5)	0.0260 (6)	−0.0050 (5)	0.0034 (5)	−0.0026 (4)
C4	0.0362 (7)	0.0171 (5)	0.0299 (6)	−0.0008 (5)	0.0012 (5)	−0.0016 (4)
C5	0.0283 (6)	0.0187 (5)	0.0272 (6)	0.0014 (5)	−0.0011 (5)	0.0006 (4)
C6	0.0219 (5)	0.0186 (5)	0.0236 (5)	−0.0010 (4)	0.0011 (4)	−0.0007 (4)
C7	0.0243 (5)	0.0181 (4)	0.0263 (6)	0.0003 (4)	−0.0021 (4)	−0.0009 (4)
C8	0.0283 (6)	0.0194 (5)	0.0258 (6)	0.0015 (4)	−0.0022 (5)	−0.0022 (4)
C9	0.0244 (5)	0.0182 (4)	0.0253 (6)	0.0022 (4)	−0.0042 (4)	−0.0015 (4)
C10	0.0217 (5)	0.0158 (4)	0.0262 (6)	0.0014 (4)	−0.0026 (4)	−0.0028 (4)
C11	0.0324 (6)	0.0178 (5)	0.0272 (6)	−0.0002 (5)	−0.0003 (5)	−0.0051 (4)
C12	0.0309 (6)	0.0174 (4)	0.0295 (6)	−0.0016 (4)	0.0018 (5)	−0.0020 (4)
C13	0.0223 (5)	0.0208 (5)	0.0251 (6)	−0.0002 (4)	−0.0007 (4)	−0.0015 (4)
C14	0.0281 (6)	0.0195 (5)	0.0264 (6)	−0.0012 (5)	−0.0032 (5)	−0.0039 (4)
C15	0.0274 (5)	0.0168 (4)	0.0275 (6)	−0.0004 (4)	−0.0045 (5)	−0.0019 (4)

### Geometric parameters (Å, °)

**Table:**

Cl1—C1	1.7383 (12)	C7—H7A	0.9300
Cl2—C13	1.7330 (13)	C8—C9	1.4775 (17)
O1—C9	1.2277 (14)	C8—H8A	0.9300
C1—C2	1.3868 (18)	C9—C10	1.4939 (18)
C1—C6	1.4024 (17)	C10—C15	1.3975 (16)
C2—C3	1.3854 (18)	C10—C11	1.3997 (17)
C2—H2A	0.9300	C11—C12	1.3857 (19)
C3—C4	1.397 (2)	C11—H11A	0.9300
C3—H3A	0.9300	C12—C13	1.3883 (18)
C4—C5	1.3800 (18)	C12—H12A	0.9300
C4—H4A	0.9300	C13—C14	1.3860 (17)
C5—C6	1.4084 (16)	C14—C15	1.3834 (19)
C5—H5A	0.9300	C14—H14A	0.9300
C6—C7	1.4574 (17)	C15—H15A	0.9300
C7—C8	1.3347 (18)		
			
C2—C1—C6	122.19 (11)	C9—C8—H8A	119.9
C2—C1—Cl1	117.81 (10)	O1—C9—C8	120.97 (12)
C6—C1—Cl1	119.93 (9)	O1—C9—C10	119.75 (11)
C3—C2—C1	119.60 (12)	C8—C9—C10	119.25 (10)
C3—C2—H2A	120.2	C15—C10—C11	118.47 (12)
C1—C2—H2A	120.2	C15—C10—C9	118.22 (10)
C2—C3—C4	119.79 (12)	C11—C10—C9	123.29 (11)
C2—C3—H3A	120.1	C12—C11—C10	120.83 (11)
C4—C3—H3A	120.1	C12—C11—H11A	119.6
C5—C4—C3	120.04 (12)	C10—C11—H11A	119.6
C5—C4—H4A	120.0	C11—C12—C13	119.13 (11)
C3—C4—H4A	120.0	C11—C12—H12A	120.4
C4—C5—C6	121.64 (12)	C13—C12—H12A	120.4
C4—C5—H5A	119.2	C14—C13—C12	121.40 (12)
C6—C5—H5A	119.2	C14—C13—Cl2	119.73 (10)
C1—C6—C5	116.74 (11)	C12—C13—Cl2	118.85 (10)
C1—C6—C7	121.68 (10)	C15—C14—C13	118.81 (11)
C5—C6—C7	121.57 (11)	C15—C14—H14A	120.6
C8—C7—C6	126.75 (11)	C13—C14—H14A	120.6
C8—C7—H7A	116.6	C14—C15—C10	121.36 (11)
C6—C7—H7A	116.6	C14—C15—H15A	119.3
C7—C8—C9	120.19 (11)	C10—C15—H15A	119.3
C7—C8—H8A	119.9		
			
C6—C1—C2—C3	0.0 (2)	C7—C8—C9—C10	163.02 (12)
Cl1—C1—C2—C3	−177.01 (11)	O1—C9—C10—C15	1.95 (18)
C1—C2—C3—C4	0.2 (2)	C8—C9—C10—C15	−176.18 (11)
C2—C3—C4—C5	−0.3 (2)	O1—C9—C10—C11	−179.57 (13)
C3—C4—C5—C6	0.2 (2)	C8—C9—C10—C11	2.31 (18)
C2—C1—C6—C5	−0.06 (18)	C15—C10—C11—C12	0.40 (19)
Cl1—C1—C6—C5	176.87 (10)	C9—C10—C11—C12	−178.08 (12)
C2—C1—C6—C7	178.80 (12)	C10—C11—C12—C13	−0.4 (2)
Cl1—C1—C6—C7	−4.27 (16)	C11—C12—C13—C14	0.09 (19)
C4—C5—C6—C1	−0.02 (19)	C11—C12—C13—Cl2	178.74 (10)
C4—C5—C6—C7	−178.88 (12)	C12—C13—C14—C15	0.16 (19)
C1—C6—C7—C8	164.24 (13)	Cl2—C13—C14—C15	−178.48 (10)
C5—C6—C7—C8	−17.0 (2)	C13—C14—C15—C10	−0.13 (19)
C6—C7—C8—C9	−179.71 (12)	C11—C10—C15—C14	−0.15 (19)
C7—C8—C9—O1	−15.08 (19)	C9—C10—C15—C14	178.41 (12)

### Hydrogen-bond geometry (Å, °)

**Table:**

*D*—H···*A*	*D*—H	H···*A*	*D*···*A*	*D*—H···*A*
C7—H7A···Cl1	0.93	2.65	3.0484 (12)	107
C7—H7A···O1	0.93	2.46	2.7973 (15)	101
C15—H15A···O1	0.93	2.46	2.7691 (15)	100
C12—H12A···O1^i^	0.93	2.59	3.2064 (16)	125

Symmetry codes: (i) −*x*+2, *y*−1/2, −*z*+1/2.
